# Thromboembolism in the left atrial appendage under atrial fibrillation and after sinus rhythm restoration: a computational study

**DOI:** 10.3389/fbioe.2026.1779828

**Published:** 2026-08-28

**Authors:** Anna Maria Lo Presti, Alessandro Tamburini, Enrico Napoli, Gaetano Burriesci, Alessandra Monteleone

**Affiliations:** 1 Ri.MED Foundation, Palermo, Italy; 2 Engineering Department, University of Palermo, Palermo, Italy; 3 UCL Mechanical Engineering, University College London, London, United Kingdom

**Keywords:** atrial fibrillation (AF), computer simulation, fluid-structure interaction (FSI), left atrial appendage (LAA), sinus rhythm (SR) restoration, thromboembolism

## Abstract

The impact of recovered atrial dynamics on pre-existing thrombi within the left atrial appendage (LAA) remains a poorly understood phenomenon, despite its potential clinical implications during spontaneous or induced restoration of sinus rhythm (SR) in patients with atrial fibrillation (AF). While computational studies have investigated thrombus formation during AF, none have examined the fate of established thrombi following SR restoration. This work addresses that gap by modelling thrombus formation and embolisation in the LAA under AF and after the return to normal rhythm. A meshless particle-based thrombus model was employed to simulate the formation of clots in a patient specific LAA, under AF conditions, and then analyse haemodynamic changes and thrombus behaviour under persistent AF and recovered SR. Results show that SR restoration profoundly modifies thrombus growth and erosion compared with AF. The resumption of atrial contractility increases local shear strain rate, especially in the proximity of anatomical features such as knees and trabeculae, driving progressive thrombi erosion and detachment from secondary lobes. These processes are amplified during the atrial systolic phase, when mechanical forces promote thrombus detachment. Conversely, if AF persists, the associated reduction in blood wash-out and shear stresses promote thrombus growth. Clots located in regions of higher stagnation remain stable under both restored SR and persistent AF. By providing the first direct computational comparison of thrombus behaviour in AF and after SR restoration, this work offers novel insights with potential to inform personalised therapeutic strategies.

## 1 Introduction

Atrial fibrillation (AF) is the most common cardiac arrhythmia worldwide (Linz et al. 2024), with a prevalence of over 50 million cases in 2021 (Tan et al. 2025). Its incidence has steadily increased over the past 2 decades (Ponamgi et al. 2021) and is projected to rise by 50% by 2050 (Lippi et al., 2021), primarily due to the continuous aging of the populations (the main risk factor), along with hypertension, diabetes, heart failure, coronary artery disease, chronic kidney disease, obesity, and obstructive sleep apnoea (Hindricks et al. 2021). Despite the reported high prevalence, the true burden of AF is likely underestimated, as a substantial proportion of cases remain asymptomatic and undiagnosed (Ponamgi et al. 2021). Silent and undetected AF significantly increases the risk of adverse outcomes, such as stroke and death (Gahungu et al. 2021). These events are linked to thromboembolism, one of the most common and severe complications of this arrhythmia (Freedman et al., 2016; Watson et al., 2009). In fact, the chaotic heart rhythm characteristic of AF promotes blood stasis, particularly within the left atrial appendage (LAA), where approximately 90% of clots responsible for thromboembolic events are reported to originate (Watson et al., 2009; Al-Saady et al., 1999).

Current guidelines (Hindricks et al. 2021) identify four different patterns of AF, based on episode duration and termination: paroxysmal (self-terminating within 7 days), persistent (lasting more than 7 days or requiring intervention), long-standing persistent (continuous for over 12 months), and permanent (accepted and untreated rhythm).

Currently, no definitive cure exists for AF, and its management relies on four pillars (Ponamgi et al. 2021): lifestyle modification to reduce risk factor, rate or rhythm control, and stroke prevention with anticoagulation or non-pharmacologic methods, such as percutaneous LAA occlusion (Landmesser et al. 2024) or LAA surgical exclusion (Ailawadi et al. 2011).

The rhythm control strategy aims to restore and maintain sinus rhythm (SR) and may involve a combination of treatments (Hindricks et al. 2021), including cardioversion (pharmacological or electrical), antiarrhythmic drugs, and catheter ablation. Cardioversion is typically indicated for haemodynamically unstable patients (Gahungu et al. 2021) and is often preceded by transesophageal echocardiography (TOE) to exclude the presence of intracardiac thrombi. This is followed by at least 4 weeks of anticoagulation to reduce the risk of thromboembolism (Ponamgi et al. 2021). In stable patients, rate or rhythm control may be considered, always alongside stroke prevention measures (Gahungu et al. 2021).

To support clinicians in evaluating thromboembolic risk and leading anticoagulation therapy decisions, clinical risk scores such as CHA_2_DS_2_-VASc are commonly used (Hindricks et al. 2021). This scoring system assigns points based on risk factors, including congestive heart failure, hypertension, age, diabetes mellitus, prior stroke, vascular disease and sex category (Hindricks et al. 2021). According to the European Society of Cardiology Guidelines (Hindricks et al. 2021), a CHA_2_DS_2_-VASc score of 0 indicates a low risk of stroke, and anticoagulation is generally not recommended; a score of 1 reflects a low to moderate risk - in these cases, the decision to initiate anticoagulation should be individualised on the base of clinical judgment and patient preferences -; a score ≥2 denotes moderate to high risk and anticoagulation is recommended. Approximately 80% of patients with AF fall into the last category and are typically prescribed anticoagulants (Ponamgi et al. 2021). Additionally, for patients with specific conditions such as severe mitral stenosis, mechanical heart valve prostheses, atrial thrombus, hypertrophic or amyloid cardiomyopathy, or congenital heart disease, anticoagulation is indicated regardless of the CHA_2_DS_2_-VASc score (Ponamgi et al. 2021). As a result, this scoring system often leads to anticoagulation recommendations for the majority of AF patients, many of whom may never experience a thromboembolic event (Ponamgi et al. 2021). More importantly, this risk score does not consider haemodynamic factors, which are crucial in thrombus formation mechanism. Specifically, according to Virchow’s triad (Virchow, 1856), blood stasis, alongside hypercoagulability and endothelial injury, constitutes a key contributor to thrombogenesis.

In particular, prolonged blood stasis promotes local accumulation of coagulation factors and activation of the coagulation cascade, leading to fibrin formation and platelet aggregation (Furie and Furie, 2008; Watson et al., 2009). This process results in thrombus formation, followed by progressive growth through the deposition of fibrin and cellular components, with increasing volume in stagnation regions (Ding et al., 2020).

In recent years, computational studies have increasingly explored the relationship between haemodynamic parameters and thrombus formation (Zhang and Gay, 2008; Koizumi et al. 2015; Otani et al. 2016; Olivares et al. 2017; Bosi et al. 2018; Masci et al. 2019; Vella et al. 2021; Musotto et al. 2022; 2024; Kjeldsberg et al. 2024; Zingaro et al. 2024). Among haemodynamic parameters, those derived from wall shear stress (WSS), such as time-averaged WSS (TAWSS), oscillatory shear index (OSI), endothelial cell activation potential (ECAP), relative residence time (RRT), and blood stasis factor (BSF), have been widely used to assess thrombus risk in the LAA (Yang et al. 2022; Chen et al. 2023; Musotto et al. 2024; Paliwal et al. 2024). Some numerical models have been developed to simulate formation and progression of thrombus in the LAA (Bouchnita et al. 2024; Lo Presti et al. 2025). Bouchnita et al. (2024) proposed a simplified model that captured the formation of wall thrombi, while Lo Presti et al. (2025) simulated thrombus growth into the LAA volume and its potential embolisation under AF conditions.

To date, no numerical models have attempted the prediction of the behaviour of pre-existing thrombi following restoration of SR. Although clinically relevant and included in current guidelines (Hindricks et al. 2021), the embolic risk in AF patients that return in SR remains poorly understood and quantified.

Abrupt changes in atrial mechanics, such as those occurring during rhythm conversion, can significantly alter flow patterns and shear stress distributions. These haemodynamic perturbations may promote surface erosion and destabilise intra-atrial thrombi (Xu et al. 2026), thereby increasing the risk of embolisation (either through complete detachment or partial fragmentation). While the occurrence of embolic events following the procedure is well-documented, direct *in vivo* evidence of thrombus fragmentation during cardioversion is currently lacking (Airaksinen et al. 2013; Garg et al., 2016; Itäinen-strömberg et al., 2024). This risk is further exacerbated in the days following SR restoration by the so-called “atrial stunning”, (Grimm et al., 1993; Dagres and Kornej, 2013; Kurt and Naqvi, 2023; Hahn et al., 2026), a phenomenon where the transient lack of effective mechanical contraction leads to low-flow states. Such prolonged stasis, occurring precisely when haemodynamics is most unstable, ensures that the embolic threat persists, or even intensifies, during the early post-cardioversion period. Clinical evidence, including TOE-confirmed cases followed by stroke, demonstrates that *de novo* thrombi can form during this window, even after the successful restoration of electrical SR (Missault et al., 1994; Tanabe et al., 1995; Kurt and Naqvi, 2023; Hahn et al., 2026).

Historically, cardioversion was permitted without prior anticoagulation in AF episodes lasting less than 48 h (Rankin and Rankin, 2017), based on the assumption of low thromboembolic risk. However, several studies have indicated that this risk is variable and influenced by patient-specific factors: Weigner et al. (1997) reported a 0.8% event rate, whereas the Fin-CV study (Airaksinen et al. 2013) showed risk ranging from 0.2% in low-risk patients to 9.8% in those with heart failure and diabetes. This evidence has prompted an update in the latest guidelines (Van Gelder et al. 2024), which now adopt a more conservative approach by reducing the window for cardioversion without prior anticoagulation from 48 to 24 h. Notably, earlier cardioversion (<12 h) remains associated with a significantly lower risk.

Although anticoagulants are effective in preventing the formation of new clots or the growth of existing ones, reducing the risk of embolic complications upon restoration of normal cardiac contraction, they are not without drawbacks. Anticoagulants increase bleeding risk and are contraindicated in patients with conditions such as severe thrombocytopenia, recent surgery or trauma, haemorrhagic stroke, or advanced liver disease (Ponamgi et al. 2021). Furthermore, although TOE is the gold standard for thrombus detection, it cannot totally exclude embolic sources. Very small, mural, or early-stage thrombi, as well as thrombotic material embedded within spontaneous echo contrast or “sludge”, may remain undetected (Melillo et al. 2019). Consequently, a residual embolic risk persists even in patients with a negative pre-cardioversion TOE.

These clinical uncertainties, together with the lack of numerical predictive models, highlight the need to elucidate the fate of intra-atrial thrombi following spontaneous or therapeutic SR restoration, not only in the absence of a preventive anticoagulation therapy, but also in patients for whom anticoagulation fails to totally eliminate the presence of thrombi.

The present study addresses this challenge by providing, for the first time, a comparative haemodynamic analysis of intra-atrial thrombi during AF and after SR restoration. Building on the thrombogenesis model developed by Monteleone et al. (2023) and previously applied to simulate thrombus formation and growth in the LAA under AF (Lo Presti et al. 2025), this study investigates thrombus behaviour throughout the process of rhythm conversion from AF to SR. The model employs a mono-physics fluid–structure interaction (FSI) framework based on the smoothed particle hydrodynamics (SPH) method, in which fluid particles meeting specific haemodynamic and biochemical conditions are converted into solids by activating elastic bonding forces (Monteleone et al. 2023). This approach enables exploration of clot erosion mechanisms and the evolving haemodynamic environment throughout rhythm conversion.

The aim of this study is to clarify the mechanisms underlying thrombus detachment, identify the anatomical regions most susceptible to embolisation, and characterise differences in thrombus behaviour between AF and SR. Ultimately, these findings provide new insights into the mechanisms potentially leading to embolic events during rhythm conversion in patients with pre-existing clots, supporting more informed and personalised clinical decision-making.

## 2 Materials and methods

### 2.1 Geometry

In this study, thrombus dynamics is analysed within a patient-specific LAA geometry previously studied in Musotto et al. (2024) and identified as “windsock” type according to the morphological classification proposed by Di Biase et al. (2012). Specifically, the geometry was directly adopted from the dataset presented in Musotto et al. (2024), where full details of image acquisition and segmentation are provided. Briefly, the model was reconstructed from computed tomography (CT) scans, acquired with a spatial resolution of 0.56 mm, of an adult subject without atrial fibrillation. The reconstructed volume corresponds to the blood pool at the beginning of the LAA filling.

**FIGURE 1:**
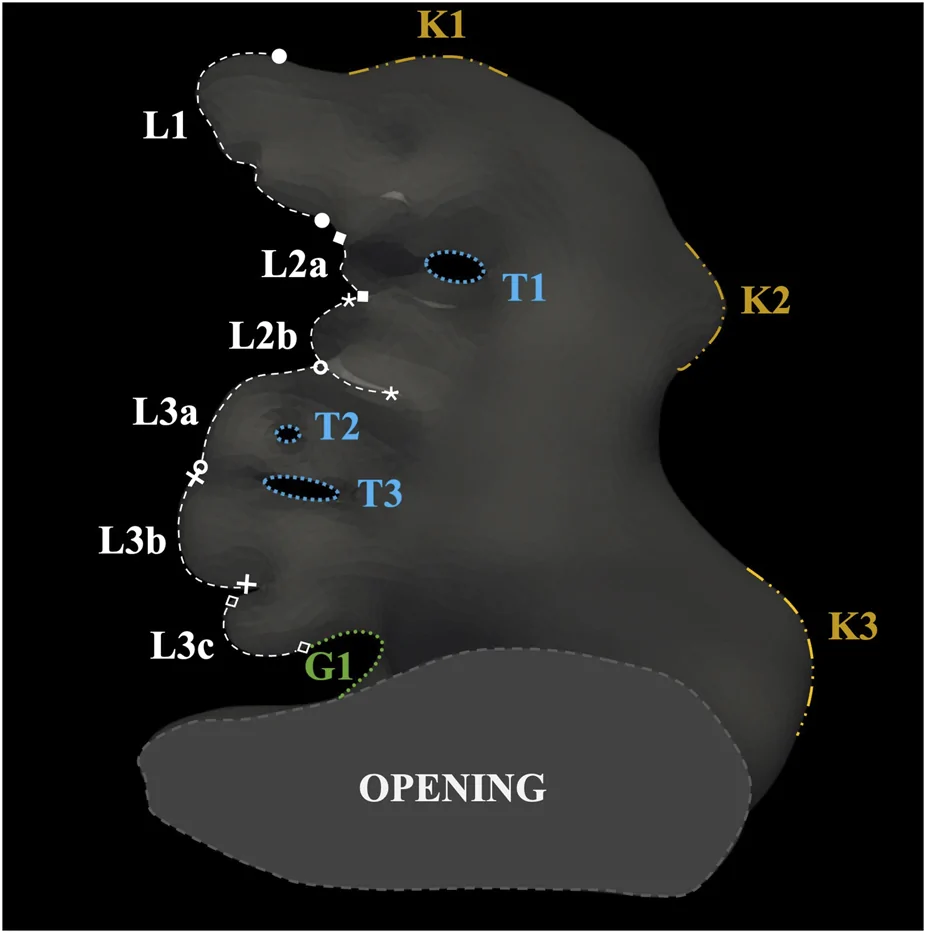
LAA features nomenclature as in Lo Presti et al. (2025), where L: lobes (dashed white lines, with markers indicating the start-end); T: trabeculae (dotted blue line); K: knees (dash–dot orange line); G: grooves (dotted green line).

As this geometry includes all main topological features that play a role in the thrombus formation process, such as lobes, trabeculae, knees and grooves (Musotto et al. 2024), the same morphology was also used in Lo Presti et al. (2025) to simulate thrombus growth in the LAA during AF. To ensure consistency and comparability with Lo Presti et al. (2025), the same nomenclature is adopted here to identify local anatomical features, with ‘L’, ‘T’, ‘K’, and ‘G’ used to indicate lobes, trabeculae, knees, and grooves, respectively (see Figure 1).

### 2.2 Numerical methodology

To evaluate thrombus evolution within the LAA, a combined numerical strategy was adopted.

**FIGURE 2:**
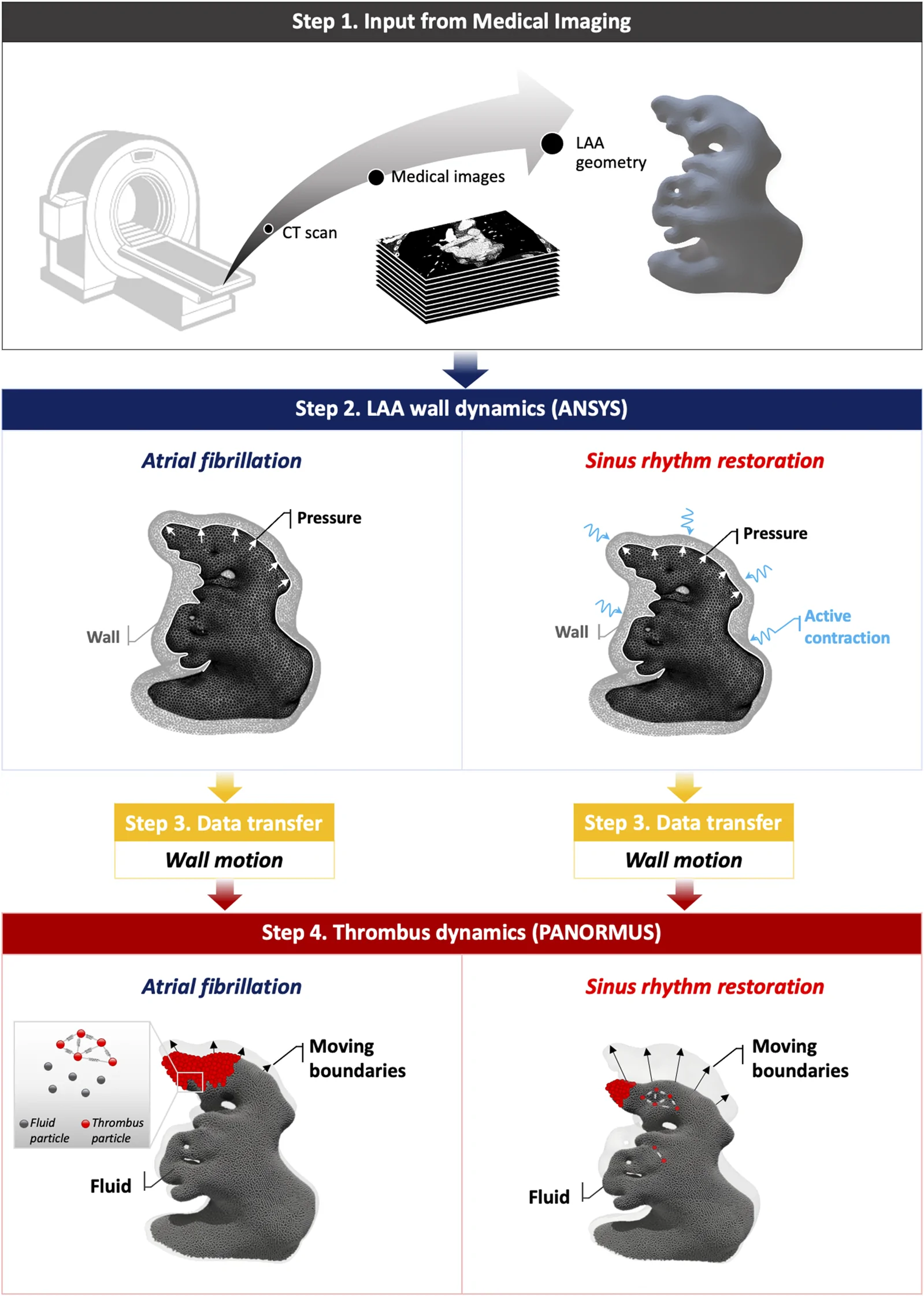
Overview of the computational workflow.

Figure 2 summarises the overall computational workflow, which integrates one-way FSI simulations performed in ANSYS 2022 R2 with mono-physics FSI simulations based on a truly incompressible SPH (ISPH) method implemented in the PANORMUS (PArallel Numerical Open-souRce Model for Unsteady flow Simulations) code (Napoli et al., 2015; Monteleone et al., 2017; Monteleone et al. 2024). Within this framework, LAA wall motion is first computed under AF and AF-to-SR transition conditions and subsequently prescribed as moving boundary conditions in the SPH solver, where thrombus formation and evolution are simulated.

A similar combined approach was previously employed in Lo Presti et al. (2025) to simulate thrombus formation under AF. In the present study, this modelling framework is adapted to investigate thrombus behaviour during rhythm conversion, with a particular focus on the haemodynamic changes occurring during the restoration of sinus rhythm.

Two simulation scenarios were considered to enable direct comparison: (i) continuous AF conditions and (ii) SR restoration, including an initial AF phase followed by transition to sinus rhythm.

In all simulations, a heart rate of 70 bpm, corresponding to a period of the cardiac cycle equal to 0.86 s, was assumed. Blood was modelled as an incompressible Newtonian fluid, a common assumption in similar computational studies (Olivares et al. 2017; Bosi et al. 2018; Musotto et al. 2024), with a dynamic viscosity of 3.7 × 10^−3^ Pa s and a density of 1062 kg/m^3^. In the simulations, blood flow is driven by LAA wall dynamics (described in the following subsection 2.2.1), while zero-pressure boundary condition was imposed at the LAA inlet/outlet, modelled as an opening (see Figure 1). This serves as a reference pressure level and, under the assumption of incompressibility, does not drive the flow.

#### 2.2.1 LAA wall dynamics

LAA wall motion was computed using the *System Coupling* module in *ANSYS Workbench* (Chimakurthi et al. 2018). The constitutive behaviour of the LAA wall was described by a third-order Ogden hyperelastic model, with parameters calibrated from uniaxial tensile tests on porcine LAA tissue (Musotto et al. 2024). Active LAA contraction and volume variation were reproduced through the virtual thermal load approach proposed by Musotto et al. (2024), in which the magnitude of the thermal load was calibrated to reproduce clinically reported human LAA filling and emptying volume variations during sinus rhythm, corresponding to approximately a 60% reduction in LAA volume during atrial emptying. Specifically, a thermal expansion coefficient of 0.03 °C^−1^ was assigned along the LAA wall surface, while a coefficient of 0.06 °C^−1^ was imposed in the out-plane-direction to enforce tissue incompressibility.

The computational domains (fluid and structural) were discretised using a tetrahedral mesh with a resolution of 70 elements/mm^3^ and a timestep of 0.0215 s, in agreement with Musotto et al. (2022) where the adequacy of this resolution has been validated.

**FIGURE 3:**
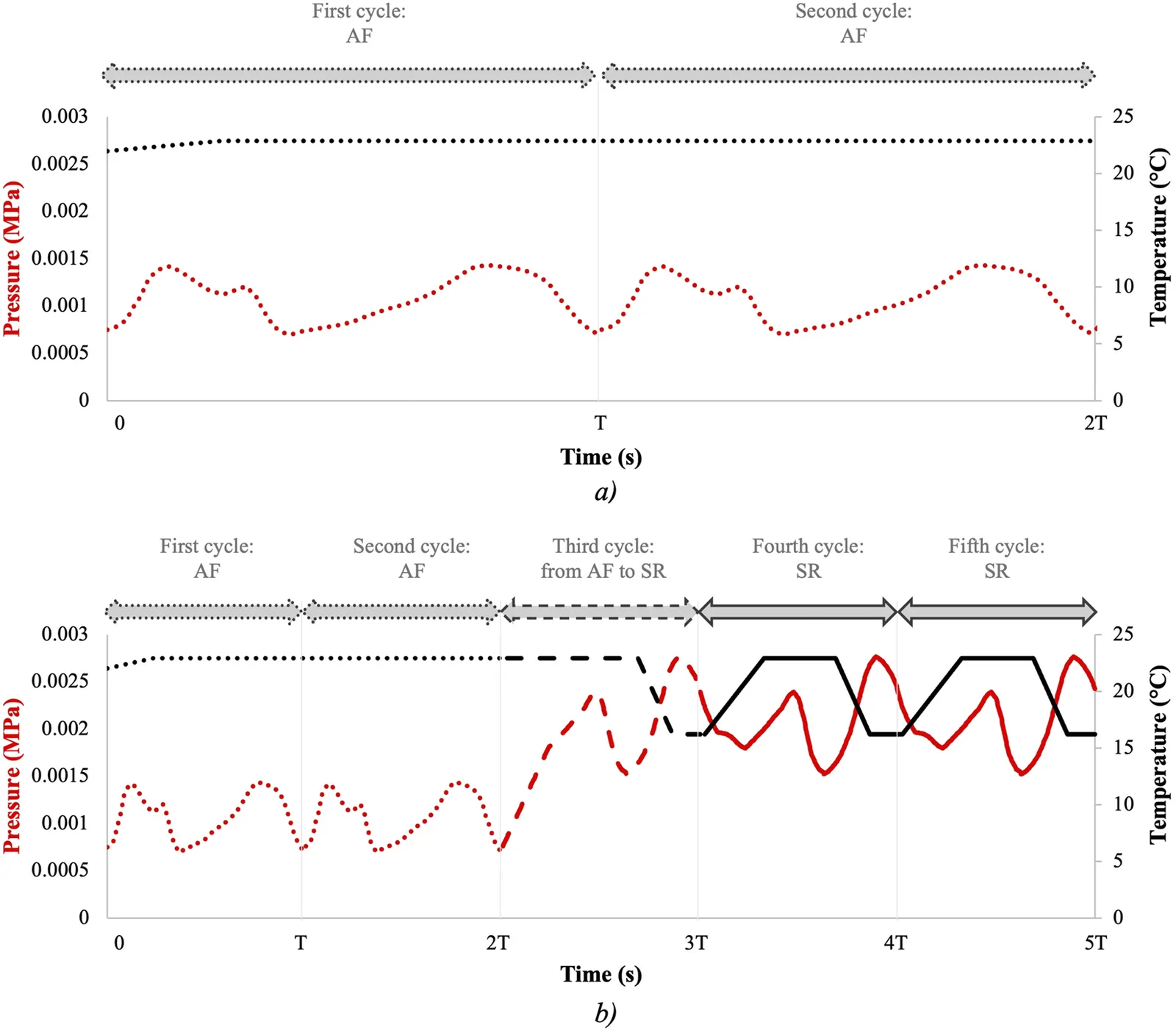
Boundary conditions used in ANSYS simulations. T = 0.86 s is the period considered for the cardiac cycle. Loads applied to the LAA structure to simulate AF **(a)** and the transition from atrial fibrillation to sinus rhythm **(b)**. Pressure and thermal curves are in red and black, respectively. Dotted lines correspond to AF, dashed lines to the transition from AF to SR, and continuous lines to SR.

AF condition was imposed through a pathological pressure waveform (dotted red line in Figure 3A), acquired from AF patients (Park et al. 2014), combined with a constant thermal load (dotted black line in Figure 3a), used to achieve the maximum physiological volume in the absence of active atrial contraction (Musotto et al. 2024).

The transition from AF to SR was simulated applying to the LAA structure a time-varying pressure waveform (red line in Figure 3b). This waveform comprises three distinct phases: (i) an initial AF phase (dotted red line), during which the pathological AF pressure profile is imposed for two cardiac cycles; (ii) a transitional phase (dashed red line), where the waveform gradually evolves towards SR over one cycle; (iii) a final SR phase (continuous red line), in which the pressure profile from healthy SR patients (Nakatani et al. 1999) is applied for two cardiac cycles - this is sufficient to achieve periodicity (Musotto et al. 2024). In addition, a thermal load (black line in Figure 3b) was applied to reproduce LAA active contraction (continuous black line), as discussed in Musotto et al. (2024).

#### 2.2.2 Thrombus dynamics

Thrombus formation and evolution were simulated using the ISPH solver implemented in PANORMUS. SPH is a Lagrangian meshless method where the computational domain is discretised into particles (Gingold and Monaghan, 1977; Lucy, 1977; Liu and Liu, 2010; Monaghan, 2012). Field variables are evaluated through kernel interpolation using a smoothing length *h*, which defines the support domain of each particle. In this study, a Wendland kernel function (Wendland, 1995) with support radius equal to 2*h* was adopted.

Within this framework, a generic field variable φ at particle position **x**ᵢ can be expressed provide Equation 2.1 as the summation over the $N_{i}$ particles lying into the support domain of i :

$$\varphi_{i}=\sum_{j=1}^{N_{i}}\frac{m_{j}}{\rho_{j}}\varphi_{j}W_{ij}$$ (2.1)

where $m_{j}$ and $\rho_{j}$ are the mass and density, respectively, of the neighbouring particle j , and $W_{ij}=W\left(x_{i}-x_{j},h\right)$ .

Incompressibility was enforced using an ISPH formulation based on the projection method of Chorin (1968), in which a predictor–corrector procedure is adopted. An intermediate velocity field is first computed neglecting pressure, and then corrected by solving a system of pressure Poisson equations, here solved using the BiCGSTAB method (van der Vorst, 1992). Further details are provided in Monteleone, Monteforte and Napoli (2017).

Wall motions derived from ANSYS simulations were imposed using the mirror particle technique (Napoli et al., 2015; Monteleone et al., 2017), based on mirroring particles located near the boundary along the wall normal direction. Specifically, to enforce the no-slip boundary condition on the moving LAA wall, the velocity of each mirror particle *m* is defined as $u_{m}=2u_{w}-u_{g}$ , where $u_{w}$ is the wall velocity (obtained from ANSYS simulation) at the intersection of the line connecting particle *m* and its generating particle *g*; and $u_{g}$ is the velocity of *g* (Napoli et al., 2015).

Simulation protocols followed the two scenarios described above. In the AF case, periodic wall motion derived from the second ANSYS cycle was imposed for all subsequent numerical cycles.

For SR restoration, the simulation included an initial AF phase of 25 cycles, during which wall motion from the second ANSYS cycle was imposed and the thrombus expanded occupying approximately 20% of the LAA volume, in agreement with Lo Presti et al. (2025). Subsequently, 1 transitional cycle (imposing the motion from the third ANSYS cycle) was run, followed by the repetition of SR cycles up to stabilisation of the thrombus volume in the LAA (with wall motion from the fifth ANSYS cycle). Thrombus behaviour was compared over the cycles following the cycle marking the transition in the rhythm, hereafter referred to as the *rhythm bifurcation*.

Transport and reactions of biochemical species involved in thrombus formation were modelled through a Lagrangian advection–diffusion–reaction framework, as previously described in Monteleone et al. (2023) and Lo Presti et al. (2025), reproducing the final stages of the coagulation cascade. The model includes seven species: prothrombin (pt), thrombin (th), fibrinogen (fg), fibrin (fi), resting platelets (rp), activated platelets (ap), and bound platelets (bp). Their transport is evaluated by solving the advection-diffusion equation (Equation. 2.2), which in Lagrangian formulation (Zhu and Fox, 2001; Herrera et al., 2009; Wang et al. 2016; Chang and Chang, 2017; Liu et al. 2020; Jiao et al. 2021; 2022; Sun et al. 2024) can be written as:

$$\frac{DC_{s}}{Dt}=\alpha_{s}\nabla^{2}C_{s}+S_{s}$$ (2.2)

with the moving coordinate system Equation 2.3

$$\frac{dx}{dt}=u$$ (2.3)

where $C_{s}$ represents the time-dependent concentration of a generic species (with s = pt, th, fg, fi, rp, ap, bp), $\alpha_{s}$ and $S_{s}$ are the diffusivity and the source term of the specie *s*, respectively, x indicates the position vector, and u the velocity vector. The material derivative (Equation. 2.4):

$$\frac{DC_{s}}{Dt}=\frac{\partial C_{s}}{\partial t}+u\cdot \nabla C_{s}$$ (2.4)

includes the convective term in the Lagrangian formulation and represents time rate change of species concentration along the path-line of a particle. Source terms, diffusion coefficients and model parameters are provided in the Supplementary Material. Further details on the solver implementation and validation can be found in Monteleone, Monteforte and Napoli (2017), Monteleone et al. (2023) and Lo Presti et al. (2025).

To accelerate the process, species diffusion was amplified by a numerical coefficient linked to the local *shear strain rate* (SSR), as described in Monteleone et al. (2023). Consequently, the simulated cycles do not temporally correspond to physiological cardiac cycles, and will hereinafter be referred to as the ‘numerical cycles’. When the concentration of bounded platelets exceeds a predefined threshold, fluid particles are converted into solid particles through elastic spring connections, whose stiffness was calibrated to reproduce the Young’s modulus of thrombus tissue. Calibration was performed by simulating uniaxial tensile tests on a solid cube, as described in Monteleone et al. (2022), and Monteleone et al. (2023). Particles can enter and leave the computational domain through the LAA inlet/outlet, following the procedure described in Monteleone et al. (2017).

The simulations were carried following the AF and SR restoration sequences described above, arresting the AF simulation at the same numerical cycle where the SR restoration sequence reached stabilisation. A constant time step of 2.15 × 10^−4^ s was used to satisfy the Courant–Friedrichs–Lewy condition and ensure numerical stability. The computational domain was discretised using Lagrangian particles with a smoothing length *h* of 5.5 × 10^−4^ m, resulting in an average particle count of 200,000 per cycle, in agreement with Lo Presti et al. (2025), where the accuracy achieved with these parameters has been validated.

## 3 Results

Stabilisation of the thrombus volume in the LAA, for the SR restoration simulation, occurred after 21 cycles from the *rhythm bifurcation*. Hence, the AF and SR restoration simulations comprise 25 initial cycles in AF conditions, which are identical for both scenarios. Afterwards, the SR restoration simulation transitions from AF to SR rhythm in one single computational cycle, and continues in SR conditions for the remaining 20 cycles. Therefore, both simulations were run for a total of 46 numerical cycles.

Below, it is provided a comparison of the behaviour in the AF and SR restoration simulations in the 21 numerical cycles following *rhythm bifurcation*, in terms of analysis of the haemodynamic changes and thrombus evolution.

### 3.1 Haemodynamic changes during AF and following SR restoration

**FIGURE 4:**
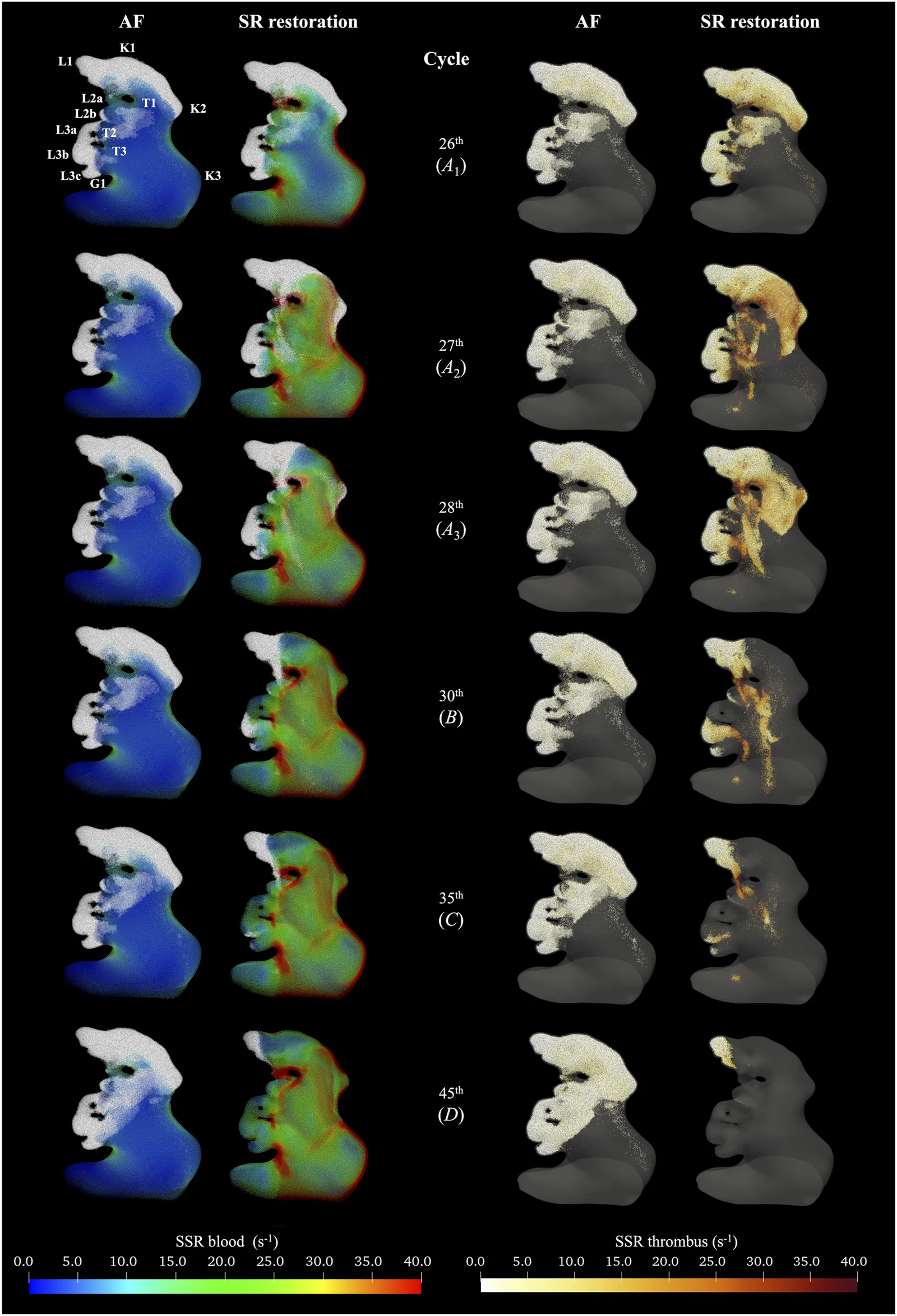
Haemodynamic changes at the instant of maximum contraction velocity of the LAA in different numerical cycles (A_1_, A_2_, A_3_, B, C and D), under AF and SR restoration conditions. **(a)** SSR of the blood particles. Solid thrombus particles are displayed with white points; **(b)** SSR of the solid particles.

Figure 4 shows the haemodynamic changes in terms of SSR, during AF and SR restoration simulations at the instant of maximum LAA contraction velocity. Results are presented across a selection of successive numerical cycles following *rhythm bifurcation*, and are indicated with capital letters (*A*_1_ = 26th, *A*_2_ = 27th, *A*_3_ = 28th, *B* = 30th, *C* = 35th, *D* = 45th). Specifically, the SSR field of blood flow is shown in Figure 4a, where thrombus particles are depicted as white spheres to emphasise the dynamic interplay between the thrombus and the surrounding fluid flow. Moreover, to better illustrate the thrombus detachment dynamics, which in Figure 4a is shadowed by the presence of the fluid particles, Figure 4b focuses exclusively on the thrombus particles, colouring them by SSR.

At the 26th cycle (*cycle A*_1_), marking the transition from AF to SR, the SR restoration case shows relatively low SSR values inside the LAA, ranging from 5 to 15 s^−1^ (see in Figures 4a,b). As the simulation advances (*cycles A*_*2*_ – *D* in Figures 4a,b), SSR increases significantly, with local peaks reaching approximately 40 s^−1^. This reflects the reestablishment of healthy haemodynamic conditions, following rhythm conversion. In contrast, SSR in the AF case remains consistently below 10 s^−1^ across the 46 simulated cycles (*cycles A*_*1*_ – *D* in Figures 4a,b).

In the SR restoration case, between the 27th and 28th cycles (*cycles A*_2_ – *A*_3_, respectively)*,* elevated SSR induces erosion of the thrombus in the region upstream of the K2 knee and near trabecula T1 (see Figure 4b). *Cycle A*_2_ in Figure 4a shows the onset of embolic potential already in the 27th cycle, with actual clot fragmentation occurring in the 28th cycle (*i.e.*, the second full SR cycle after the transition phase). This follows sustained exposure to high SSR, in the range 20–25 s^−1^, as shown in *cycle A*_2_ in Figure 4b. In subsequent cycles (see *cycles B* - **C** in Figures 4a,b), the clot previously located near the K2 knee has completely left the LAA, while thrombi in lobes L3a and L2b experience similar erosion due to persistent SSR values of 20 s^−1^ or higher. By the 35th cycle (*cycle C*), a substantial volume of thrombotic material remains within the mid-region of the LAA, influencing the fluid field significantly, as evident in Figure 4a. In contrast, in the AF case, consistently low SSR levels (*cycles A*_2_ –*C* in Figures 4a,b) promote the growth of the clotted region, which expands to the trabeculae T2-T3 and the central region.

In the following cycles of the SR restoration case (see *cycle D* in Figures 4a,b), residual thrombi located in lobes L3b and L3c are progressively eroded, leaving only the thrombus at the LAA tip, which remains stable throughout the simulation. This region experiences consistently low SSR values, below 5 s^−1^, despite restoration of healthy sinus rhythm. By contrast, in the AF case, thrombus growth continues, eventually occupying about one-third of the LAA volume (*cycle D* in Figures 4a,b).

**FIGURE 5:**
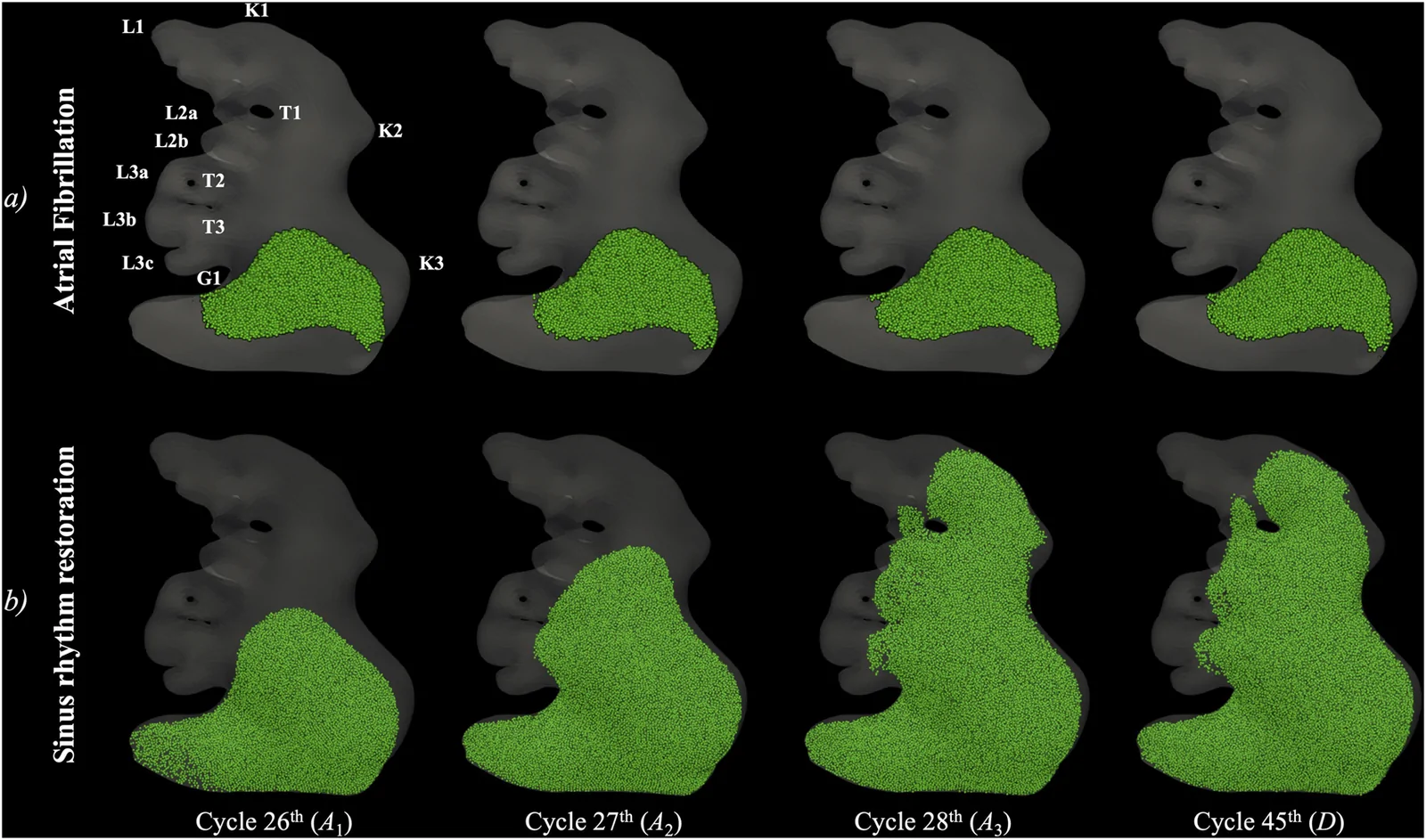
Distribution of newly entered blood particles (green points) having residence time less than one cycle. Instant of maximum expansion velocity of the LAA at the A_1_, A_2_, A_3_ and D numerical cycles under: **(a)** AF; **(b)** SR restoration.

Figure 5 shows the distribution of newly entering fluid particles, defined as those that have spent less than one numerical cycle within the modelled domain, for the AF and SR restoration scenarious (panels *a* and *b*, respectively). Again, the analysis focuses on the final 21 numerical cycles following *rhythm bifurcation*, at the instant of maximum expansion velocity of the LAA, considering the sequence of numerical cycles introduced in Figure 4. Under AF conditions, these new particles remain confined near the LAA opening throughout the simulation (see *cycles A*_1_ – *D* in Figure 5a). In contrast, after SR restoration (*cycles A*_1_ – *D* in Figure 5b), the new particles progressively penetrate deeper into the appendage. Specifically, at the 26th cycle (*cycle A*_1_), corresponding to the AF-SR transition, they reach about halfway into the LAA; by the 27th cycle (*cycles A*_2_), they extend just beneath trabecula T1. From the 28th cycle onward (*cycles A*_3_ – *D*), particles advance beyond T1 and approach the K1 knee region, although they do not irrorate the tip or secondary lobes.

### 3.2 Dynamics of thrombus particles during AF and post SR restoration

**FIGURE 6:**
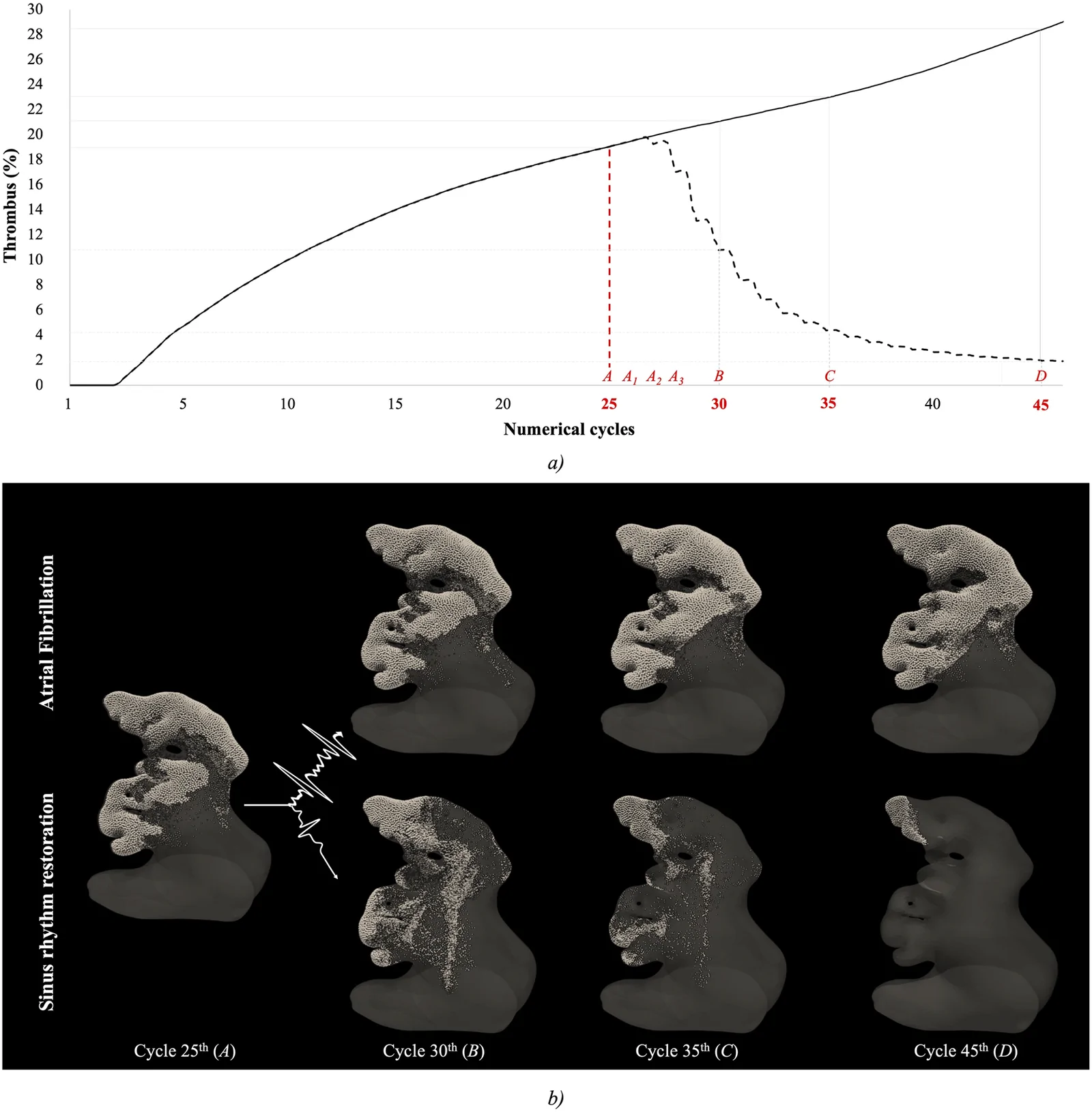
**(a)** Graph of the thrombus evolution over time. Continuous black line: AF simulation; dashed black line: SR restoration simulation. **(b)** Thrombus configuration, represented by beige spheres, at the A, B, C and D numerical cycles, at the instant of maximum expansion velocity of the LAA, under AF and SR restoration conditions.

Figure 6a depicts the temporal evolution of thrombus, defined as the percentage of solid particles relative to the total number of particles within the domain, for both AF (continuous black line) and restored SR (dashed black line). Figure 6b shows thrombus configurations at the 25th (*cycle A* marked with a dashed red line in Figure 6a), 30th (*cycle B*), 35th (*cycle C*) and 45th (*cycle D*) numerical cycles. Thrombus configurations are shown at the instant of maximum LAA expansion velocity, for both AF and SR restoration cases. Of note, since *cycle A* precedes the *rhythm bifurcation*, its resulting thrombus configuration is identical for both AF and SR restoration simulations.

Up to the 25th cycle (*cycle A* in Figure 6a), the two curves overlap, reaching ∼ 19% of thrombus formation (about one-fifth of LAA volume), as both cases are under identical AF conditions. During the transitional cycle (*cycle A*_1_ in Figure 6a, *i.e.* 26th) and the first half of the subsequent fully established SR cycle, thrombus percentage remains nearly identical (see Figure 6a). From the latter part of the 27th cycle onward (*cycle A*_2_ in Figure 6a), AF conditions shows a gradual increase in solid particles fraction, whereas in the SR restoration case the clot volume progressively decreases (see Figures 6a,b), with the largest reduction (approximately 4%) occurring between the 28th (*cycle A*_3_ in Figure 6a) and 29th cycles. In the subsequent cycles, the decrease becomes slower: around 2% between the 30th (*cycle B* in Figure 6a) and 31st cycles, less than 2% from the 32nd cycle onward, and below 1% from the 34th cycle, with progressively smaller variations. Within each cycle, percentages remain relatively stable during diastole and decline during atrial systole. This phase-dependent behaviour becomes less evident after the 38th numerical cycle, as the quantity of solid particles exiting the domain becomes marginal. From the 42nd cycle, thrombus volume stabilises, reaching a plateau by the 45th cycle (see *cycle D* in Figure 6a), with residual thrombus remaining at the LAA tip (see *cycle D* in Figure 6B).

**FIGURE 7:**
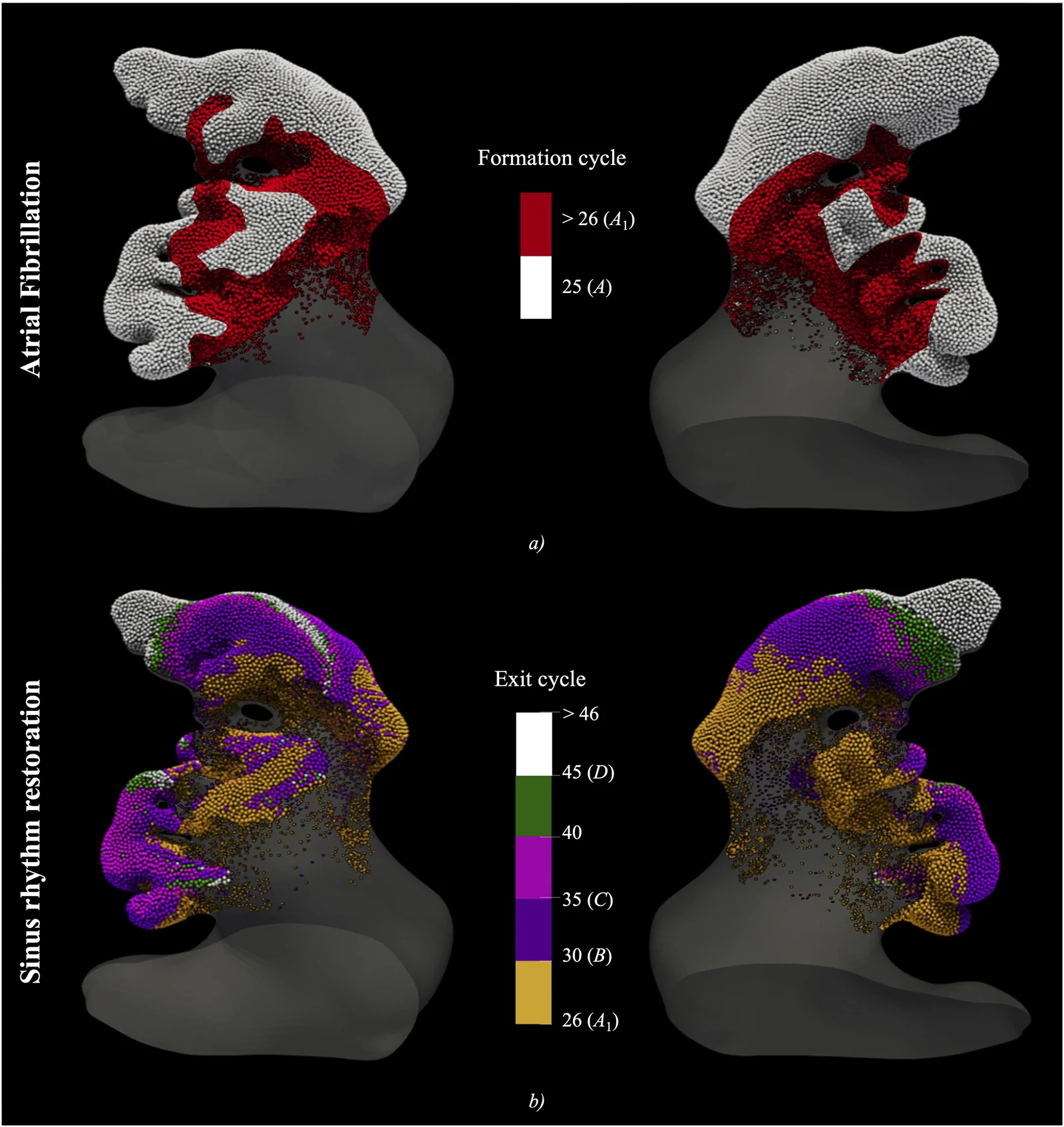
**(a)** Newly formed map of thrombus solid particles in AF. Solid particles at the 25th cycle (white points); new thrombus particles formed during 26th–46th cycles (red points); **(b)** exit map of solid particles in the SR restoration case. Solid particles leaving the domain at: 26th-30th cycles (orange points); 31st-35th cycles (purple points); 36th-40th cycles (magenta points); 41st-45th cycles (green points). Solid particles that remain within the domain beyond the 46th cycle (white points). Front and back views are shown for both maps.

Figures 7a,b shows, respectively, the distribution of newly formed thrombus solid particles in the AF case and the exit map of solid particles in the SR restoration case. Specifically, Figure 7a depicts the thrombus present at the 25th AF cycle (*i.e., cycle A*) as white particles, while the new solid particles formed over the following 21 cycles following *rhythm bifurcation* (26th–46th) are represented in red. As highlighted above, these new clotted regions mainly form in the proximity of trabeculae T2–T3 and in the central region of LAA. As concerns SR simulation, Figure 7b shows the thrombus at the 25th cycle (*i.e., cycle A*), prior to rhythm change, with each solid particle coloured by the numerical cycle in which it exits the domain, thus mapping the embolised material that progressively enters the left atrium after SR restoration. The majority of solid particles (approximately 8%) exit the domain between the 26th (*i.e., cycle A*_*1*_) and 30th (*i.e., cycle B*) cycle (orange points). Particles near knee K2 and trabeculae T2-T3 exit between the 31st and 34th cycle (purple points). Particles from the secondary lobes (L3a, L3b and L3c), together with those around knee K1, leave between the 35th (*i.e., cycle C*) and 40th cycle (magenta points). A small fraction near the LAA tip embolises between the 41st and 45th cycle (green points). As noted above, thrombus located at the tip of the LAA and even thrombi near lobe L3a and knee K1 (white points) permanently remain inside the domain for the entire simulation.

## 4 Discussion

The combined analysis of Figures 4–7 offers a comprehensive view of the complex haemodynamic mechanisms governing thrombus evolution, fragmentation, and embolisation under AF and SR restoration, particularly in the absence of preventive anticoagulation therapy. Under AF conditions, SSR remains low (generally below 10 s^−1^), limiting thrombus erosion and promoting the accumulation of new solid particles, mainly near trabeculae T2–T3 and in the central region of LAA (Figures 4, 6, 7a).

In contrast, SR restoration induces a marked rise in SSR within the LAA, transitioning from the low values typical of AF to substantially higher more physiological levels as atrial contractility resumes. Anatomical regions such as knee K2 and trabeculae T1-T3 exhibit SSR peaks up to 20–40 s^−1^, creating fluid-mechanical conditions that promote thrombus erosion and fragmentation (see Figure 4). This increase in SSR also enhances blood washout, evidenced by progressive influx of newly entering fluid particles that penetrate deeper into the LAA after SR restoration (see *cycles A*_1_ – *D* in Figure 5b). The washout flow, which is largely absent from distal LAA regions during AF (*cycles A*_1_ – *D* in Figure 5a), produces high shear stresses at the thrombus interface (see Figure 4) which facilitate progressive thrombus dislodgement. This leads the sequential disappearance of thrombus material from lobes L2a, L2b, L3a, L3b and L3c. The temporal pattern of thrombus reduction closely follows the atrial systolic phase, indicating that atrial contraction exerts mechanical forces which promote thrombus detachment. Importantly, unlike thrombus formation and growth, which involve the continuous deposition of platelets and fibrin and therefore occur over a relatively long time period (Teeraratkul et al. 2024), embolisation is associated with a rapid asportation process that occurs within seconds (Xu et al. 2017). Results show that approximately 20 numerical cycles are necessary to increase the percentage of solid particles by 50% under AF conditions. In the case of SR restoration, thrombus masses released into the circulatory system are substantial from the first instants after SR recovery, with about 50% of the clot expelled in the first six cycles, and then progressively stabilising. This suggests that ischaemic risks after spontaneous or induced rhythm restoration are immediate if a thrombus occupying a significant percentage of the LAA volume is present.

Conversely, small thrombi located in sheltered regions of the LAA exhibit markedly higher stability throughout the simulation (see white points in Figure 7). These regions are characterised by persistent flow stagnation and low SSR values, typically below 5 s^−1^. This value is coherent with the threshold recommended by Musotto et al. (2024) to identify the regions of unphysiologically low persistent shear rates, where red blood cell aggregation is expected to be promoted. The limited penetration of new fluid particles into these anatomical niches creates a protective microenvironment, effectively insulating the local thrombus from the mechanical stresses driving erosion in other regions. Though, it needs to be underscored that the adopted model does not describe the physiological thrombolytic mechanisms, as it does not considering the fibrinolytic system. Therefore, a thrombus exhibiting apparent stability within the present simulations may progressively be reabsorbed. Still, the simulation suggests low risk of ischaemia associated with small distal clots.

It is important to note that, in this work, thrombus fragmentation is governed by local haemodynamic stresses acting at the blood–thrombus interface. Elevated shear stresses following SR restoration preferentially act on the outer, less compact layers of the thrombus, promoting boundary erosion and progressive detachment, in agreement with the mechanism described by Xu et al. (2026).

Overall, results highlight the heterogeneous nature of thrombus dynamics within the LAA, where local haemodynamic conditions play a decisive role in determining the thrombus fate during SR restoration.

## 5 Limitations

The interpretation of the present findings should consider several limitations. In particular, the analysis was performed on a single patient-specific LAA geometry. Given the large morphological variability of the LAA, local flow patterns, thrombus location, and entrapment mechanisms may differ across patients. Therefore, the present results should be interpreted as a demonstration of the mechanisms underlying thrombus destabilisation under the investigated conditions, rather than as a population-based quantification of thromboembolic risk. While qualitative mechanisms governed by local haemodynamic features, such as shear gradients and stagnation zones, are expected to be preserved across different morphologies, quantitative thresholds, spatial patterns, and thrombus release dynamics are likely to be geometry-dependent.

Moreover, the transition from AF to SR was represented as an idealised restoration of atrial mechanical activity over one cardiac cycle. Although both AF and SR haemodynamic conditions were derived from clinical measurements, patient-specific data describing the recovery of atrial mechanical function immediately after cardioversion are currently unavailable. Consequently, the model does not reproduce the progressive recovery of atrial contractility associated with atrial stunning. Simulating such gradual recovery over the clinically relevant time scale of days to weeks (Khan, 2003) would require resolving a prohibitively large sequence of numerical cardiac cycles within the proposed computational framework. Therefore, the present study considers an idealised recovery scenario in which sinus rhythm contractile function is restored immediately following rhythm conversion, allowing the mechanical effects of restored atrial contraction on a pre-existing thrombus to be isolated. Consequently, the simulations are intended to isolate the biomechanical consequences of restored atrial contraction and should not be interpreted as predicting the clinical time course of thrombus embolisation after cardioversion.

Further, thrombus mechanical behaviour was simplified. The clot was assumed to exhibit homogeneous material properties, without accounting for spatial variations in composition or degree of organisation, and the adopted erosion criterion has not yet been validated against experimental measurements. Therefore, the predicted fragmentation dynamics should be interpreted qualitatively, with emphasis placed on the underlying haemodynamic mechanisms rather than on absolute erosion rates.

The model does not include biological processes such as fibrinolysis. The simulations focus on the early mechanical response of a pre-existing thrombus to haemodynamic changes induced by restored atrial contraction during the early post-cardioversion period (days, Dagres and Kornej, 2013), whereas fibrinolytic mechanisms act over substantially longer timescales (weeks to months, Killewich et al., 1997). *In vivo*, fibrinolysis would likely contribute to progressive thrombus dissolution, particularly in regions characterised by persistent low shear. Accordingly, the model may overestimate long-term thrombus persistence in these regions.

Blood was modelled as a Newtonian fluid, thereby neglecting the non-Newtonian behaviour of blood, which becomes more pronounced in regions of persistently low shear rates. This choice follows Musotto et al. (2024), who demonstrated negligible differences between Newtonian and Casson rheological models under haemodynamic conditions comparable to those investigated here. Accordingly, the Newtonian assumption was considered sufficiently accurate for the objectives of the present simulations.

Finally, thrombus formation was computationally accelerated. Therefore, numerical cycles do not correspond directly to physiological time and should not be interpreted as predictors of the clinical timing of thrombus formation or release from the LAA. However, because only the diffusion coefficients of the modelled biochemical species were artificially enhanced, the computed haemodynamic fields (e.g., velocity and shear strain rate) remain unchanged by the temporal acceleration. Consequently, although the simulated cycles cannot be mapped directly onto physiological time, the mechanistic interpretation of the shear-driven thrombus destabilisation process is preserved.

Despite these limitations, the present study provides, to the best of our knowledge, a first mechanistic framework to investigate how haemodynamic changes during rhythm restoration influence thrombus stability and release from the LAA.

## 6 Conclusion

This study investigates the behaviour of thrombus within the LAA during AF and following the SR restoration, in the absence of preventive anticoagulation therapy.

Under AF conditions, low SSR values limit thrombus erosion, driving to progressive expansion of the thrombus regions.

Following SR restoration, SSR levels increase markedly with the resumption of atrial contraction, contributing to the mechanical erosion and detachment of thrombus fragments. The embolisation process primarily affects the thrombi located in the central cavity and in the secondary lobes, with the largest reduction in solid particle presence already observed in the first cycles after SR is restored. The thrombus located at the most distal regions, however, may remain stable, and eventually be reabsorbed. This persistence is associated with permanently low local SSR and long residence times, even when SR is restored. Analysis of blood washout through newly entering fluid particles further confirms that this region is poorly perfused, remaining largely isolated even under restored haemodynamic conditions.

Overall, the simulations clearly indicate that restoration of SR in the presence of a clot of relevant size (in the analysed case, occupying about one-fifth of the LAA volume) can lead to an immediate and widespread potential embolisation of thrombotic material. On the other hand, small clots in distally peripheral regions (such as the tip of the LAA) may be tolerated, as the washout remains ineffective also in SR, allowing their stable permanence until they are, eventually, metabolically reabsorbed. These findings underscore the embolic risk associated with rhythm conversion in patients with AF-related thrombi and reinforce the importance of preventive anticoagulation during this critical clinical phase. Importantly, this work provides the first direct computational evidence comparing thrombus dynamics in AF and after SR restoration, offering novel insights into clot fate during rhythm conversion.

## Data Availability

The original contributions presented in the study are included in the article/Supplementary Material, further inquiries can be directed to the corresponding authors.
