# Supplementary material for: Thromboembolism in the left atrial appendage under atrial fibrillation and after sinus rhythm restoration: a computational study
Source: Front Bioeng Biotechnol. 2026 Aug 28;14:1779828. doi: 10.3389/fbioe.2026.1779828 (PMC13562033; doi:10.3389/fbioe.2026.1779828)
Supplement: Supplementary file 1 [file Table1.docx]

***Supplementary material***

*Table S1: Reaction kinetics and source terms*

| Reactions | Source terms | Kinetic constants |
| --- | --- | --- |
| 1. *Thrombin generation*  $\boldsymbol{rp+pt}\boldsymbol{\to th}$  $\boldsymbol{ap+pt}\boldsymbol{\to}\boldsymbol{th}$ | $S_{th}=k_{th}^{rp} C_{rp} C_{pt}+k_{th}^{ap} C_{ap} C_{pt}$  $S_{pt}= -S_{th}$ | $k_{th}^{rp}=$6.50∙10^-10^ U PLT^-1^ s^-1^ μM^-1^  (Sorensen *et al.* 1999)  $k_{th}^{ap}=$3.69∙10^-10^ U PLT^-1^ s^-1^ μM^-1^ (Sorensen *et al.* 1999) |
| 2. *Fibrin generation*  $\boldsymbol{fg}\boldsymbol{+th}\boldsymbol{\to}\boldsymbol{fi}$ | $S_{fi}=\frac{k_{fi}^{th} C_{th} C_{fg}}{K_{m,fi}^{th}+C_{fg}}$  $S_{fg}= -S_{fi}$ | $k_{fi}^{th} =$59 s^-1^  (Anand, Rajagopal and Rajagopal, 2003)  $K_{m,fi}^{th}=$3160 nM  (Anand, Rajagopal and Rajagopal, 2003) |
| 3. *Platelet activation*  $\boldsymbol{rp}\boldsymbol{\to}\boldsymbol{ap}$  $\boldsymbol{th}\boldsymbol{\to}\boldsymbol{ap}$ | $S_{ap}=k_{ap} C_{rp}+k_{ap}^{th}\phi_{ap}^{th***}C_{rp}$  $S_{rp}=-k_{ap} C_{rp}{-k}_{ap}^{th}\phi_{ap}^{th}C_{rp}$ | $k_{ap}= \left\{ \begin{aligned} 0, \Omega<1 \\ \frac{\Omega^{*}}{{t_{act}}^{**}}, \Omega\geq1 \end{aligned} \right.$  (Sorensen *et al.* 1999)  $k_{ap}^{th}=$ 0.5 s^-1^  (Kuharsky and Fogelson, 2001) |
| 4. *Clot formation*  $\boldsymbol{ap}\boldsymbol{+fi}\boldsymbol{\to}\boldsymbol{bp}$ | $S_{bp}=k_{bp} \phi_{bp}^{fi****}C_{ap}$  $S_{ap}=-k_{bp} \phi_{bp}^{fi****}C_{ap}$ | $k_{bp}=$10^4^ s^-1^  (Leiderman and Fogelson, 2011) |

*pt: Prothrombin, th: Thrombin, fg: Fibrinogen, fi: Fibrin, rp: Resting Platelets, ap: Activated Platelets, bp: Bound Platelets, k: Rate constant, S: Source terms*

**Notes**

^*^ $\Omega=\sum_{s = 1}^{N_{s}} w_{s}\frac{C_{s}}{C_{s}^{*}}$

represents a function of local agonist concentrations used to evaluate the combined effect of different chemical species on platelet activation (Sorensen *et al.* 1999). Here, $w_{s}$is the weighting factor associated with species $s$*,* $C_{s}$is its local concentration, and $C_{s}^{*}$ is the corresponding threshold concentration.

In the present model, platelet activation is driven exclusively by thrombin, and therefore:

$$\Omega=w_{th}\frac{C_{th}}{C_{th}^{*}}$$

with$w_{th}= 1$ and $C_{th}^{*}$= 9.11$\cdot$10^-10^ M (Sorensen *et al.* 1999).

^**^ $t_{act}=$1 s denotes the time required for platelet activation (Sorensen *et al.* 1999).

^***^ $\phi_{ap}^{th}=\frac{C_{th}^{4}}{C_{th}^{4}+C_{th,50}^{4}}$

is a fourth-order Hill function used to describe cooperative binding effects in thrombin-mediated platelet activation

(Sarrami-Foroushani *et al*., 2019). The parameter $C_{th,50}$ corresponds to the half-saturation concentration, defined as the thrombin concentration at which half-maximal activation occurs (Ingalls, 2013) and it is equal to $C_{th}^{*}$= 9.11$\cdot$10^-10^ M (Sorensen *et al.* 1999).

^****^ $\phi_{bp}^{fi}=\frac{C_{fi}^{2}}{C_{fi}^{2}+C_{fi,50}^{2}}$

is a second-order Hill function describing cooperative binding in fibrin-mediated platelet adhesion (Ingalls, 2013). The parameter $C_{fi,50}$ represents the fibrin concentration corresponding to half-maximal binding (Ingalls, 2013) and is set to 600 nM (Sarrami-Foroushani *et al*., 2019).

*Table S2: Diffusion coefficients*

| Species | Diffusion coefficients | Reference |
| --- | --- | --- |
| *pt* | $\alpha_{pt}=$ 5.21$\cdot$10^-7^ cm^2^ s^-1^ | (Anand, Rajagopal and Rajagopal, 2003) |
| *th* | $\alpha_{th}=$ 6.47$\cdot$10^-7^ cm^2^ s^-1^ | (Anand, Rajagopal and Rajagopal, 2003) |
| *fg* | $\alpha_{fg}=$ 3.1$\cdot$10^-7^ cm^2^ s^-1^ | (Anand, Rajagopal and Rajagopal, 2003) |
| *fi* | $\alpha_{fi}=$2.47$\cdot$10^-7^ cm^2^ s^-1^ | (Anand, Rajagopal and Rajagopal, 2003) |
| *rp* | $\alpha_{rp}=$ 2.5$\cdot$10^-7^ cm^2^ s^-1^ | (Leiderman and Fogelson, 2011) |
| *ap* | $\alpha_{ap}=$ 2.5$\cdot$10^-7^ cm^2^ s^-1^ | (Leiderman and Fogelson, 2011) |
| *bp* | $\alpha_{bp}=$ 0 cm^2^ s^-1^ | (Sarrami-Foroushani *et al*., 2019) |

*Table S2: Initial concentrations*

| Species | Initial concentrations | Reference |
| --- | --- | --- |
| *pt* | $C_{pt\_0}=$ 1400 nM | (Sarrami-Foroushani *et al*., 2019) |
| *th* | $C_{th\_0}=$ 0 nM | (Sarrami-Foroushani *et al*., 2019) |
| *fg* | $C_{fg\_0}=$ 7000 nM | (Sarrami-Foroushani *et al*., 2019) |
| *fi* | $C_{fi\_0}=$ 0 nM | (Sarrami-Foroushani *et al*., 2019) |
| *rp* | $C_{rp\_0}=$ 2$\cdot$10^8^ PLT ml^-1^ | (Sarrami-Foroushani *et al*., 2019) |
| *ap* | $C_{ap\_0}=$ 1$\cdot$10^7^ PLT ml^-1^ | (Sarrami-Foroushani *et al*., 2019) |
| *bp* | $C_{bp\_0}=$ 0 PLT ml^-1^ | (Sarrami-Foroushani *et al*., 2019) |

**References**

Anand, M., Rajagopal, K., and Rajagopal, K. R. (2003). A Model Incorporating Some of the Mechanical and Biochemical Factors Underlying Clot Formation and Dissolution in Flowing Blood. Journal of Theoretical Medicine 5, 183–218. doi: 10.1080/10273660412331317415

Ingalls, B. (2013). Mathematical modeling in systems biology: an introduction. MIT Press.

Kuharsky, A. L., and Fogelson, A. L. (2001). Surface-Mediated Control of Blood Coagulation: The Role of Binding Site Densities and Platelet Deposition. Biophys J. 80, 1050–1074. doi: 10.1016/S0006-3495(01)76085-7

Leiderman, K., and Fogelson, A. L. (2011). Grow with the flow: A spatial-temporal model of platelet deposition and blood coagulation under flow. Mathematical Medicine and Biology 28, 47–84. doi: 10.1093/imammb/dqq005

Sarrami-Foroushani, A., Lassila, T., Hejazi, S. M., Nagaraja, S., Bacon, A., and Frangi, A. F. (2019). A computational model for prediction of clot platelet content in flow-diverted intracranial aneurysms. J. Biomech. 91, 7–13. doi: 10.1016/j.jbiomech.2019.04.045

Sorensen, E. N., Burgreen, G. W., Wagner, W. R., and Antaki, J. F. (1999). Computational Simulation of Platelet Deposition and Activation: I. Model Development and Properties. Ann. Biomed. Eng. 27, 436–448. doi: 10.1114/1.200
